# An exploratory research on the role of family in youth's drug addiction

**DOI:** 10.1080/21642850.2014.939088

**Published:** 2014-08-20

**Authors:** Sobia Masood, Najam Us Sahar

**Affiliations:** ^a^ Department of Behavioral Sciences, Fatima Jinnah Women University, Rawalpindi, Pakistan

**Keywords:** drug addiction, Pakistani youth, rehabilitation, parental involvement, emotional expressiveness, family therapy in Pakistan

## Abstract

Most of the researches in Pakistan are concerned with the aetiological factors of drug addiction among the youth. However, few studies seek to explore the social aspects of this phenomenon. The present study aimed to explore the role of family, the influence of parental involvement, and communication styles in youth's drug addiction in a qualitative manner. Twenty drug addicts (age range 18–28 years) were taken as a sample from drug rehabilitation centres in Rawalpindi and Islamabad, Pakistan. A structured interview guide was administered comprising questions related to the individual's habits, relationship with family and friends, and modes of communication within the family. Case profiles of the participants were also taken. The rehabilitation centres offered family therapy and the researcher, as a non-participant, observed these sessions as part of the analysis. The demographic information revealed that majority of the participants were poly-substance abusers (80%) and the significant reasons for starting drugs were the company of peers and curiosity. The thematic analysis revealed parental involvement and emotional expressiveness as two major components in family communication. It was found that parents were concerned about their children, but were not assertive in the implementation of family rules. It was also found that the major life decisions of the participants were taken by their parents, which is a characteristic of collectivist Pakistani society.

## 1. Introduction

Drug abuse is a prevalent problem among Pakistan's youth, who account for 28% of the whole population of Pakistan (Niaz, Siddiqui, Hassan, Ahmed, & Akhtar, 2005; Royen & Sathar, 2013; United Nations Office on Drugs and Crime [UNODC] (2013)). According to the recent report by UNODC and Pakistan Bureau of Statistics in 2013, an estimated 6.45 million of the population in Pakistan use drugs on an annual basis with cannabis being the most commonly used drug.

Among the youth population, nearly 25% are involved in some form of drug abuse. Among the youngest drug users, between the ages of 15 and 19 years, the most commonly used drug is cannabis. Compared to other national estimates, opiate use is very high with one million people using heroin or opium. The use of ‘Sheesha’^1^ with cannabis (charas) and other drugs is a new emerging trend and is being abused by both males and females, mostly from the upper socio-economic strata living in the posh areas of cities (Ministry of Narcotics Control, Islamabad, Year Book, 2012).

Availability of substances such as cannabis is determined culturally. The community and government influence and decide which drugs should be controlled and how. Under Pakistani law, shop owners cannot legally sell drugs, even cigarettes, to individuals below 18 years of age, but people tend to find ways to get around the law. In a number of studies of substance abuse in the developing world, drug use has been characterized by the use of low-priced and accessible drugs, such as cannabis, alcohol, and tobacco, and volatiles, such as glue (United Nations International Children's Emergency Fund, as cited in Sherman, Plitt, Hassan, Cheng, & Zafar, 2005).

The effects of substance abuse have far-reaching consequences. They not only affect the user him/herself, but also their families, and society as a whole. The work sector loses able-bodied individuals, which in turn affects the economy. The family's role in the development of substance abuse is unique as the family simultaneously suffers from the direct consequences of the abuse, while also holding the potential to be one of the most powerful protective influences against it.

In supporting efforts to control drug-related problems in the country, the rehabilitation centres in Pakistan mostly focus on culturally adapted rehabilitation techniques. Common therapies used in rehabilitation centres include a modified version of the Alcoholics Anonymous treatment and family therapy. Most of these rehabilitation centres are present in the urban areas and they recruit psychologists and psychiatrists to work in these facilities.

Multiple drug use among young people is now a widespread phenomenon prevalent in almost all sections of society. A large number of young people, both male and female, experiment with a variety of illegal substances such as cannabis products (such as Hash/Hashish and marijuana), Ketamine,^2^ cocaine, and heroin. Drug abuse among the youth of Pakistan is becoming a major issue, and identifying the family dynamics and interfamily communication styles that may influence a youth to turn to drugs is the target of this article.

### 1.1. Family dynamics

Pakistan is a collectivistic culture in which the family is given priority as social and financial support is coveted. Family is the core for need fulfilment. This is why joint families prevail in which grandparents, parents, children, and their uncles and aunts all live together. Members make major life decisions with the approval from elders of the family as they are the primary support network.

Family dynamics can be defined as the way a family is structured including the individual interpersonal roles played by the members within the family unit. Family dynamics is the basis for all individuals to learn how to cope with the challenges they might face in later life. Parental support and unconditional positive regard strengthen self-esteem and self-confidence, and their absence reduces them.

Drug abuse is often referred to as a family issue because of the serious negative consequences of addiction and because the importance of recovery affects not only the substance abuser, but also all the members of the family. Therefore a focus on the role of families is critical in understanding and preventing the destructive intergenerational cycle of substance abuse and addiction.

According to the findings of Bahr, Maughan, Marcos, and Li's (1998) study, the parent–adolescent bond has indirect effects through religiosity, and family drug use. It was found that among family variables the two major variables were for bond to mother, followed by family drug problem. Bond to father, parental monitoring, and family aggression were relatively weak predictors of adolescent drug use. Since the bond to mother is stronger, adolescents feel closer to them and share their daily life routine, thus communicating frequently with their mothers. In Pakistani society a strong bond to mother is observed as fathers are seen as the authority figure. Poor communication within the family unit affects an individual's indulgence in drug abuse.

The findings from Manley, Searight, Skitka, Russo, and Schudy's (1991) study found that the families of adolescent drug abusers were more reserved in their expression of thoughts and feelings. As fathers in Pakistani society are responsible for discipline, they are seen as less warm and as such communicating on a one-on-one basis can prove to be a little difficult. Out of the many conflicts within a family, the lack of problem-solving abilities, interactions, and communication are related to further addiction (Hosseinbor, Bakshani, & Shakiba, 2012; Sajida, Zia, & Irfan, 2008).

The objective of this study was to explore the role of family, the influence of parental involvement, and communication styles in youth drug addiction.

## 2. Methodology

### 2.1. Participants

The participants included in the study were substance abusers seeking treatment from validated rehabilitation centres. The participants were selected through purposive sampling technique from rehabilitation centres which were offering family therapy sessions, and where the clients were residing in the facilities for a minimum of three months in order to facilitate detoxification. All the participants were males between the ages of 18 and 28 years old and were being treated in various drug rehabilitation centres in Rawalpindi and Islamabad, Pakistan.

The present research was conducted on 20 individuals who had an understanding of their dependency. A preliminary study was conducted which helped in building rapport with the participants. It also helped the researcher in identifying the inclusion and exclusion criteria. Participants were excluded from the study if they were in an active phase of mental disorders such as bipolar disorder, depression, or stable schizophrenia.

The facilities from which the data were collected were offering family therapy sessions conducted by trained psychologists. These sessions were attended by immediate family members of the individuals admitted for substance abuse. The family members included in the therapy sessions were mostly the parents and wives of the clients. These sessions were attended by the researcher with permission from the facility as well as the family members.

### 2.2. Instruments

#### 2.2.1. Demographic data sheet

A demographic data sheet was used to collect data regarding the age, family income, family system, siblings, marital status, family system, familial illnesses, as well as data regarding the substance abused, frequency of abuse, relapses, and the beginning of the addiction (Table 1).

**Table 1.** Frequency and Percentage of participants demographic variables (age, education, marital status, family system, birth order, occupation, mother, and father).

Variable	Label	Frequency (*f*)	Percentage (%)
Age	18–22 years	3	15.0
	23–28 years	17	85.0
Education	Uneducated	2	10.0
	Up to fifth grade	5	25.0
	Up to tenth grade	9	45.0
	College level	4	20.0
Marital status	Single/unmarried	9	45.0
	Married	8	40.0
	Separated/divorced	2	10.0
	Widowed	1	5.0
Family system	Joint	15	75.0
	Nuclear	5	25.0
Birth Order	First born	2	10.0
	Middle born	14	70.0
	Last born	4	20.0
Occupation	Employed	14	70.0
	Unemployed	6	30.0
Mother	Alive	18	90.0
	Deceased	2	10.0
Father	Alive	12	60.0
	Deceased	8	40.0

Note: (*N* = 20).

#### 2.2.2. Interview guide

A structured interview guide was constructed consisting of questions referring to the individual's habits, relationship with family and friends, modes of communication within the family as well as the social aspects of their lives. The interview guide was adapted and extended from the Family Functioning Style Scale by Deal, Trivette, and Dunst (1988) and Dunst, Trivette, and Deal (1988).

#### 2.2.3. Observation technique

It was a practice in the rehabilitation centres to hold family therapy sessions. The family therapy sessions were conducted by three consultant psychologists of the facility, at different times, and were attended by three to five members of the participant's family, which included the parents and spouses. Consent was obtained from the psychologists and the attending family members for the researcher to attend the sessions herself as a non-participant observer. The data collected from therapy sessions were in the form of field notes.

Additionally, informal discussions were held with the participants, the psychologists, and some of the family members present in the facility during the preliminary study. These discussions helped the researcher in rapport-building and in understanding the setting of the session.

The researcher made case profiles of the individuals which helped in formulating a comprehensive picture of the particular individual and in the corroboration of the information collected in the focus group discussions during the family therapy sessions (Table 2).

**Table 2.** Frequency and percentage of participants according to demographic variables of drug use, duration of drug use, single substance or multiple substance abuse, number of treatments, and reasons for starting drugs.

Variables	Label	Frequency (*f*)	Percentage (%)
Drug use	Single	4	20.0
	Multiple	16	80.0
Duration of abuse	Less than a year	0	0
	1–5 years	3	15.0
	6–10 years	8	40.0
	>10 years	9	45.0
Substance abused	Single	4	20.0
	Multiple	16	80.0
No. of treatments received	First	6	30.0
	1–5 times	13	65.0
	6–10 times	0	0
	11–15 times	0	0
	16–20 times	1	5.0
Reasons for drugs	Single	10	50.0
	Multiple	10	50.0
Multiple reasons reported by the respondents	Friends	5	25.0
	Accident	1	5.0
	Curiosity	4	20.0

Note: (*N* = 20).

### 2.3. Procedure

Data were collected from rehabilitation centres which were sanctioned by the Anti-Narcotics Force of Pakistan in the vicinity of Islamabad and Rawalpindi. Some government and private institutions offer family therapy along with rehabilitation of drug addicts which is why these specific centres were selected. The interviews took a minimum of 30–40 minutes each. The data were collected over a period of four weeks in the form of notes. Research ethics were followed as the researcher took consent from the participant in the form of signatures on a consent form.

The data collected were then translated from the native language, Urdu, into English. The themes were generated based on the common responses by participants.

The researcher used the interpretive paradigm to code the verbatim responses collected from the participants. A master code sheet was created on the basis of themes identified in Family Functioning Style scale (Dunst et al., 1988). The researcher continued to code the responses while conducting interviews and observing the interaction between family members during the sessions. Consensus was reached through committee approach during analysis.

Coding was applied to the responses pertaining to communication styles, social relationships, and family dynamics. Subcategorization was done based upon the patients' responses and then counterchecked with the psychologists and families. The coding was then subjected to a peer review process which involved the researchers and two anthropologists in order to facilitate inter-coder reliability. (Table 3)

**Table 3.** Case studies.

Initials	Age	No. of treatments	Reason given
Mr Z.A.	21	1st	Peers
Mr S.	26	1st	Death of elder brother
Mr F.	21	1st	Curiosity, peers
Mr T.	25	1st	Peers
Mr M.A.	23	2nd	Peers, free time
Mr A.A.	28	2nd	Peers
Mr S.A.M.	28	1st	Curiosity
Mr R.W.	28	2nd	Peers
Mr SM	28	2nd	Peers
Mr A.	27	1st	Peers
Mr R.U.	28	1st	Authoritarian father, psycho-social stressors, victim of child abuse
Mr N.M.	23	4th	Due to injury sustained in an accident
Mr K.	28	3rd	Peers
Mr A.	27	1st	Peers
Mr S.S.	22	1st	Peers
Mr N.	23	1st	As a mistake
Mr D.	28	19th	Curiosity
Mr R.	26	3rd	Peers
Mr N.I.	28	4th	Peers

## 3. Results

The numerical data regarding the demographic characteristics showed that the majority of the participants (85%) were within the age range of 23–28 years; 45% were unmarried, 40% were married, 10% were separated or divorced, and 5% were widowed. It was found that majority of the participants (75%) lived in a joint family system, while 25% lived in a nuclear family system. The majority of the participants (45%) were educated up to and above the tenth grade, and all the participants were living with their parents. According to the demographics, both parents of the majority (60%) of the participants were alive.

The drug-related questions revealed that the majority of the participants (80%) were poly-substance abusers, and 45% had been abusing drugs for over 10 years. A relapse ratio was estimated by the number of treatments received by individuals from various facilities. Of the participants, 65% had received treatments 1–5 times, 30% were being treated for the first time, and 5% had received treatments 19 times, indicating a high rate of relapse which can be linked to dysfunction within the family that can be seen in the following analysis. One respondent, who had relapsed three times and was in the facility for his fourth treatment, stated that he had relapsed because of fights with his father.

Referring to the reasons which caused the participants to become involved in substance abuse, 50% cited a single reason while the other 50% stated multiple reasons, of which the company of peers (indicating peer pressure), curiosity, and stress were commonly cited.

### 3.1. Thematic analysis

In analyzing the responses and information provided by the participants and verified by family members and psychologists during family therapy sessions, the following themes and related subcategories presented themselves.

#### 3.1.1. Parental involvement

This theme contains information about family dynamics in terms of functions performed by all members, as well as information concerning family cohesion, communication patterns, familial support, decision-making, parental control, and supervision, among other topics.

*Spending time with family members*. Spending time with family is important. The majority of participants (80%) stated that they communicated frequently with their parents on a regular basis, i.e. every day, and whenever they had time to do so. As one respondent specifically stated:

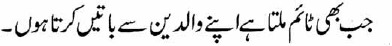

Whenever I get the time I talk to my parents

Only 20% of the participants stated that they seldom communicated with their parents. A majority (70%) of the participants stated that they spent more time with their mothers rather than their fathers, e.g. a respondent stated:

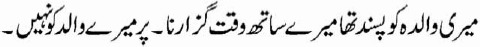

My mother liked spending time with me. My father didn't.

Over half (55%) of the participants stated that they were closer to their mother than their father, which can be explained through the cultural context as in Pakistan fathers are reserved in their affections towards their children.

*Mode of communication with parents*. Another sub-theme was the mode of communication with parents, 55% of the participants stated that they communicated with both parents on a one-on-one basis. In relation to communicating with the father, 15% stated that they did communicate with their father on one-on-one basis, but also used another family member as mediator (usually the mother). One of the reasons given by the participants was that the fathers were not willing to listen to the individuals; hence they approached the mother, e.g. a respondent stated:

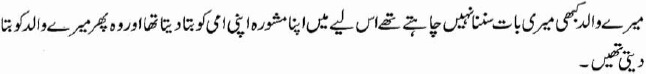

My father never bothered to listen to what I had to say. So I told my mother my opinion and she later conveyed it to my father.

This statement explored the problems in family communication which was further explored and the following reasons were reported by the participants. One of the reported barriers to effective communication is the refusal to talk. Though the parents were not reported to ignore their children, 60% of the participants reported that when they were angry, the parents stopped communication in order to avoid further conflict.

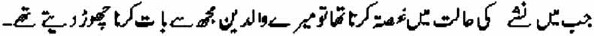

When I became angry while being intoxicated, my parents stopped talking to me.

The reasons given for arguments were not agreeing on certain things and being beaten, along with siblings, by the father, e.g. a respondent stated:

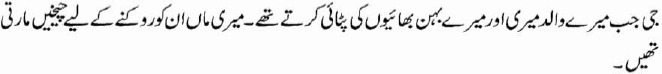

Yes, when my father used to beat me and my siblings, my mother used to scream at him to top hitting us.

Out of the total participants, 65% reported no incidence of arguments involving yelling with parents. However, 35% of them reported frequent arguments with fathers. Moreover, they reported their fathers yelling at them frequently. During an interview, a respondent stated:

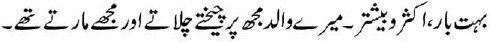

Frequently, my father used to yell at me, hit and scream at me

*Range of issues being discussed with parents*. All the participants were aware of the prescribed family rules such as mobility, social circles, and expenditures; hence the breaking of the rules incited the parents' disapproval. This can be connected to the parents' attempt at controlling the negative behaviour of the participants. Almost all the participants agreed upon the activities disliked by their parents such as the use of drugs, coming home late, not listening to their parents' advice, spending money on drugs, selection of friends, refusal to go to school, and poor academic performance. However, it was found that these rules were not put into practice because the parents were not assertive in their implementation.

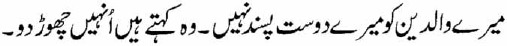

My parents don't like my friends. They tell me to leave them.

Most of the participants reported a consensus on major decisions such as choice of life partner, career, and academics. Seventy per cent of the participants agreed that their parents knew how they felt and thought about any given situation.

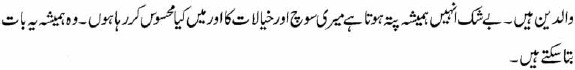

They're parents. Of course they know what I'm thinking and feeling. They can always tell

Pertaining to the communication links between the individuals and their family, 50% of the participants stated that they could discuss anything with both their parents. They were given the freedom to express their opinions and their advice was sought. Only 15% of the participants stressed that they discussed issues more with their father than their mother, another 15% of the participants stated that they used to discuss their problems with both of the parents before being involved in substance abuse, but afterwards they were more comfortable discussing their problems with an elder sibling (usually a brother) as evident from the following statement:

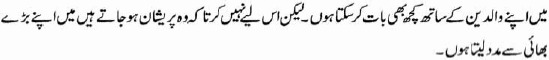

I can discuss every problem with my parents. I don't because then they'll get worried. So I go to my elder brother for guidance.

Referring to the parents' approval of the friends of the participants, a majority of the participants (65%) stated that their parents did not approve of their peers as they feared that their children would get into bad habits such as substance abuse.

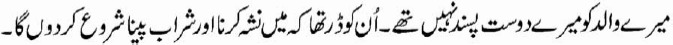

My father never liked my friends. He was afraid I would get into bad habits like substance abuse or drinking alcohol.

Pertaining to parents' knowledge of their whereabouts, 75% of the participants reported that their parents were aware.

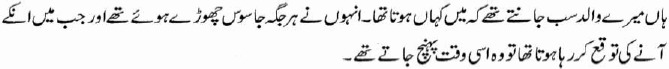

Yes, my father knew where I was. He had spies everywhere. He used to drop by when I least expected him to, to check up on me.

Of the participants, 15% stated that their parents had no idea about their child's social life. A respondent, upon being asked about his parent's knowledge of his whereabouts, reported:

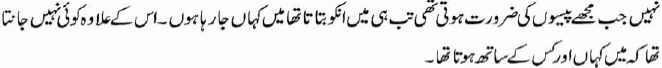

No. Only when I needed money from them did I tell them where I was going. Otherwise no one knew where I was or with whom.

The choice of selecting their own life partner is an important aspect where communication discrepancies can be brought to the surface. It can also be one of the major causes of drug addiction as reported by participants. In a collective culture like Pakistan, life partner selection is mostly done by the family as the family provides financial, social, and moral support. This is why most people prefer arranged marriages. In the present study, out of the 40% of married participants, two-thirds of them had arranged marriages. One of the advantages of an arranged marriage is social compatibility, where both families work together to keep harmony. Of the total participants, 75% reported that their opinion on family issues were held in regard and respected. They stated that their parents were ultimately responsible for making all the decisions.

All participants were focused on their future goals, which included quitting drugs, getting married, reuniting with their spouses, and finding secure jobs to support their families. The decision-making is influenced by family support and it is this social support which helps in future planning.

*Emotional expression*. In this section the participants reported the characteristics of their communication styles with their families. The data within this section help in exploring the gaps perceived by the participants in communicating with their families which could possibly lead to conflict and may provide insight into their addiction.

*Using profanities*. Sixty per cent of the participants admitted to using profane language generally, which is one of the common characteristics of problematic communication style. Thirty-five per cent of them admitted to using profane language with their family, but only under the influence of drugs or alcohol, e.g. a respondent stated:

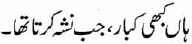

Yes [I curse] sometimes. When I was under the influence of drugs I used to curse.

They used profanities in conversations with their friends as well as in response to situations. The rest of the participants denied using profane language.

*Being good listeners & speakers*. All participants agreed that they were good listeners with whom family and friends could discuss various problems. They also felt that they were good speakers, being clear in their expression and maintaining eye contact. However, it was observed that 15% of the respondents mumbled while conversing with the researcher.

*Assertiveness*. A majority of the participants reported themselves to be assertive and expressive; however, discrepancies were found amongst the statements given by the participants pertaining to communication with their parents and friends. The participants stated outright that they were assertive, that they were able to say ‘no’ to a decision taken by their family, and that they took responsibility for their actions. However, upon conversing with the researcher, some participants negated this statement. As recorded, a participant, upon being asked if he expressed his feelings in regard to a given situation, stated:

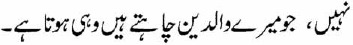

No. My parents make all the decisions.

*Expression of negative emotions and arguments*. Ten per cent of the participants stated that they used drugs as a means to express themselves. They viewed substance abuse as an escape from reality. Upon being asked how they expressed their negative emotions, most of the participants (80%) stated that they yelled and shouted.

*Presenting Nervous Tics*. Forty-five per cent of the participants reported nervous tics such as nail biting, smoothing hands over their lips, and playing with their hair. This might be due to the side effects of the drugs or the medication used in the treatment. One of the reasons stated by a participant was that because he used heroin in *panni* (silver paper), the skin under his nails blackened and thus biting his nails became a habit.

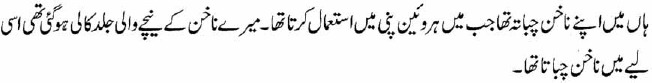

Yes, I used to bite my nails. That was when I used to use heroin in panni. The skin under my nails used to be blackened hence I bit my nails out of habit.

The aforementioned analysis revealed parental involvement and emotional expressiveness as two major components in family communication. It has been further revealed that culture plays an important role in family dynamics with reference to the major life decisions being taken. Another aspect identified was the presence of physical abuse in the family. With reference to the communication style, it has been noted that the elements of respect and emotional expressiveness have been present. This will be further discussed in the following section.

## 4. Discussion

It was found by the researchers that all the participants unanimously agreed in their communication styles stating that they were assertive, good listeners, and future-oriented. However, it was found that there were discrepancies among the participants' views and the observations of the researcher and the psychologists involved in their rehabilitation. The participants sometimes refused to make eye contact and were not clear in their verbal expression, i.e. they mumbled answers, which may be due to the effect of the medications they are given for their treatment.

It is the understanding of the researcher, as well as the consulted psychologists, that these discrepancies can be attributed to denial. In the case of drug abuse, impaired insight causes what is referred to as ‘denial’. Denial of addiction is a common, if not a core, feature of most substance-use disorders and has been conceptualized as a psychological phenomenon (Rinn, Desai, Rosenblatt, & Gastfriend, 2002). The first step of treatment is the confrontation of this denial, as reported by the psychologists.

The findings of the present research are consistent with previous researches which stated that the family of a drug addict plays an important role as the causal or aetiological factor for the addiction itself. The main problem reported was with authoritarian fathers and submissive mothers, as well as lack of communication between parents and children, particularly with their fathers and during conflicts. This has been reflected in the thematic analysis under the subsection of spending time with family members. It can be inferred from other studies that authoritarian parents, who are highly demanding but less responsive, tend to make demands on their children but not respond well to their needs. It can also be inferred that due to this parenting style a communication gap can develop among the family which prohibits direct communication, especially the expression of anger (Verdejo-Garcia, Rivas-Perez, Vilar-Lopez, & Perez-Garcia, 2007).

Parental monitoring has been associated with elements of parental control such as imposing rules and restrictions on children's activities and associations (Borawski, Ievers-Landis, Lovegreen, & Trapl, 2003; Nash, McQueen, & Bray, 2005). Monitoring of adolescents' behaviour, which includes tracking and surveillance, is an essential parenting skill. A large amount of studies show that well-monitored youths are less involved in delinquency and other norm-breaking behaviours (Cleveland, Feinberg, Osgood, & Moody, 2012; Stattin & Kerr, 2000). The findings of the present study are in contradiction with the findings of the aforementioned studies as 75% of the participants stated that their parents were aware of their whereabouts, and yet, the participants were still involved in norm-breaking behaviours. One of the possible reasons for this can be the lessened mobility restrictions on males in Pakistani culture.

It was observed by the researcher and the psychologists that dysfunction within the family of an addict was the leading cause of relapse. Lavee and Altus (2001) also noted that individuals in a dysfunctional family were at a higher risk of relapse than those who were not. It was found by the researcher that most of the participants (65%) who had relapsed did so because of the problems they faced within their family, for example communicating directly with the fathers who were unwilling to listen and talk to their sons. This, in the case of 35% of the participants, resulted in frequent arguments with their fathers which involved yelling.

In addition to the family being the aetiological factor in addiction, it was found paradoxically to be part of the cure as well. Repeated admissions to the rehabilitation centres show continued family support since the families were willing to invest emotionally and financially in the improvement of their sons. In Pakistan choice and financial liability are on parent's part, so this practice can be used as an indicator of family support in the treatment.

Since the family plays an integral part in the rehabilitation process, the centres included the component of family therapy into the treatment programme. This will help to integrate the family and improve communication between the individuals as well as encourage families to show their support to the addict. Through their support the family can lessen environmental stressors such as peer pressure and help the addict.

It can be stated that if parents and significant others identify the signs of addiction at an early stage, the family can stage an intervention and potentially stop the behaviour before it becomes worse. The importance of communication between the parents (especially fathers) and children is stressed as it may be the key link in the detection and prevention of self-destructive acts.

### 4.1. Conclusion

The current study provides an insight into the role played by family in youth's drug addiction. It found that parental involvement and emotional expressiveness were two of the major themes identified.

### 4.2. Limitations and suggestions

The present study aimed to explore the role of family in youth drug addiction, but there are a few limitations of the study that must be taken into consideration.

First is the time limit. The researcher did not have ample time to conduct the study on a larger number of participants because of the short time period supplied for this project. Due to this shortage of time, the time spent on one-on-one session with the participants was limited. Hence unstructured interviews and a deeper exploration into the problems stated by the participants were not possible.

The present study is focused only on the role of parents in youth addiction, not taking into consideration the role of siblings or peers of the addicted. However, the researcher believes that if the study were to be conducted in a longitudinal manner, more aetiological factors would come to light for addiction with respect to Pakistan. Other areas of interest as well as other factors, bio-socio-cultural factors, remain to be explored as well as their contribution to addiction among youth.

## Notes

1. An oriental tobacco pipe with a long flexible tube connected to a container where the smoke is cooled by passing through water.

2. Ketamine – commonly used as a safe, effective, anesthetic agent, but misused as a club drug.
